# University Experience during the First Two Waves of COVID-19: Students’ Experiences and Psychological Wellbeing

**DOI:** 10.3390/ejihpe13080108

**Published:** 2023-08-11

**Authors:** Valentina Lucia La Rosa, Elena Commodari

**Affiliations:** Department of Educational Sciences, University of Catania, Via Biblioteca 4, 95124 Catania, Italy; e.commodari@unict.it

**Keywords:** university, education, COVID-19, risk perception, psychological wellbeing

## Abstract

Background: This study aimed to analyze Italian university students’ psychological needs, learning experiences, and wellbeing during the first two waves of the COVID-19 pandemic. Methods: The study was carried out during the first wave of the pandemic, and then during the second. A total of 1230 university students (654 in stage 1 and 576 in stage 2) completed a battery of validated questionnaires exploring students’ experiences in distance learning, perceived health risks related to COVID-19, and psychological wellbeing. Results: MANOVA showed a significant main effect of the pandemic stage on students’ learning experience and wellbeing. In particular, students were more distracted and concerned about their academic careers during the second phase of the pandemic than in the first. Furthermore, the pandemic stage also significantly affected health risk perceptions and fear of COVID-19, which were higher during the second wave of the pandemic. Female gender and concern for a university career were significant risk factors for high levels of negative affectivity and low levels of positive affectivity during the pandemic. In contrast, adherence to COVID-19 restriction measures and good family support were protective factors. Conclusions: It is essential to promote adequate university psychological services to support university students who have experienced the adverse psychological effects of the pandemic and enhance the resilience factors needed to improve their wellbeing in the post-pandemic period.

## 1. Introduction

### 1.1. Background

For young people, the university experience is an important stage in the individual life cycle, since the start of college studies coincides with the transition phase that opens the door to adulthood and independence [1]. The changes that a student’s lifestyle inevitably undergoes after the end of high-school studies, often accompanied by the abandonment (albeit in many cases temporary) of the family home to move to university campus cities, have a significant impact on the sense of self and identity, as well as on psychophysical wellbeing [2,3]. Furthermore, changes in daily life management and teaching–learning modes, in addition to affecting personal and professional socio-relational dynamics, seem to have peculiar physiological effects expressed in increased brain sensitivity to stress [4].

Therefore, it seems clear that, even in the absence of para-normative and unexpected events, the transition to college life, while fostering in the student the development of a sense of belonging that can improve academic wellbeing and performance [4], can also be stressful and characterized by feelings of uncertainty and fear for the future [5,6]. In this regard, university students’ psychological wellbeing has long been a topic of study. In particular, numerous studies have pointed out that university students often experience mental health problems such as depression and anxiety [7]. Students’ academic performance and social functioning may be adversely affected by these problems, which may also affect their career prospects in the future [8,9]. This particular vulnerability that seems to characterize the experience of university students can be exacerbated by unforeseen events that threaten the regularity of the academic career and lead, in many cases, to withdrawal from the university [4].

In this sense, the COVID-19 pandemic can be considered to be a para-normative event that significantly affected the experience of university students around the world. In fact, from the early stages of the pandemic, universities closed, and the university life of millions of students was dramatically transformed [10]. Due to the lockdown measures, university students had to modify and adapt their study methods to distance learning [11,12], and they experienced fear and uncertainty for their personal and professional future [13,14]. Furthermore, new rules were needed to deal with post-lockdown university life, which inevitably changed university students’ educational processes and sociality [15].

### 1.2. Theoretical Contribution

Several studies on the impact of the COVID-19 pandemic on university students reported adverse psychological effects, including post-traumatic stress symptoms, depression, anxiety, and loneliness [16,17,18,19]. Specifically, most students with high levels of anxiety and depression during the pandemic mainly reported difficulties related to online education, uncertainties about their university career, feelings of social isolation, and concerns about future job prospects [15,16]. In this regard, a study at the University of Heidelberg (Germany) [20] confirmed that 72.2% of the university students surveyed felt that their wellbeing was seriously impaired. In particular, they reported severe depressive symptoms and anxiety. Furthermore, they cited loneliness, depression, and lack of recognition as their most significant complaints during the pandemic [20]. Similar results were also obtained from Wang et al.’s study [21] on 2,031 US college students, showing moderate-to-severe anxiety and depression during the pandemic.

Browning et al. [16] aimed to identify the main risk factors for adverse psychological consequences among university students during the pandemic. According to their results, being a woman, having a poor general health status, being 18 to 24 years old, spending eight or more hours on the screen daily, and knowing someone infected predicted higher levels of psychological distress. Similarly, the study by Islam et al. [22] on a sample of 476 university students living in Bangladesh underlined that females and older students reported higher levels of anxiety and depression.

However, these results are not entirely generalizable, because university students in each country experienced different conditions related to varying trends in infections and various restrictive measures imposed by governments on daily and academic life. Therefore, it is essential to conduct studies considering the context in which university students live to obtain valuable results, identify risk and protective factors, and adequately support students in coping with the profound changes produced by the pandemic. Furthermore, it is also essential to explore the long-term impacts of the pandemic and the risk and protective factors concerning the psychosocial implications of this event.

### 1.3. Aims of the Study

Based on these considerations, this study aimed to explore the needs, learning experiences, and psychological wellbeing of a large sample of Italian university students during the first two waves of the COVID-19 pandemic. More specifically, our objectives were (a) to compare the experiences and the degree of satisfaction with distance learning during the first and second waves of the pandemic, (b) to measure the dimensions of health risk perception related to COVID-19 during the first two waves of the pandemic, and (c) to analyze the psychological wellbeing of the university students in our sample over the course of a year.

## 2. Materials and Methods

### 2.1. Study Design and Participants

This cross-sectional study is part of a larger research project on the psychosocial impact of COVID-19 on university students during the different stages of the pandemic.

Specifically, the first stage covered the initial period of the COVID-19 pandemic (between April 22 and May 1, 2020, during the first lockdown in Italy). An original, anonymous, and voluntary questionnaire was distributed online to Italian university students through the main social networks. The questionnaire was proposed again during the later stages of the pandemic, characterized by varying degrees of viral spread and restrictive measures. The data reported in this study referring to the second wave of the pandemic (between April and May 2021) were compared with those for the first lockdown, already published in a preliminary study conducted by our group [23].

All participants were informed about the study’s objectives, methodology, and estimated duration, and they completed an online informed consent form before completing the questionnaires. The study protocol adhered to the ethical standards of the Declaration of Helsinki and the Ethics Code for Italian Psychologists (L. 18.02.1989, n. 56), the Italian Law for Data Privacy (DLGS 196/2003), and the Ethics Code for Psychological Research (March 27, 2015) approved by the Italian Psychologists Association. The Internal Review Board of the Department of Educational Sciences of the University of Catania approved the study protocol.

### 2.2. Measures

The battery of validated questionnaires used in this study consisted of the following sections:

– *Sociodemographic section:* Sex, age, type of university course, residence in the university town, residence in a “red zone” (an area of Italy where the government has imposed stricter measures of containment due to exponential and uncontrolled growth in contagion cases compared to other regions of the country), number of persons in the household, and whether they had contracted COVID-19.
– *Distance learning*: Students were asked to answer a series of items related to their experience of distance education during the pandemic and its impact on their academic performance (e.g., “In this period when I have to stay at home when I study, I get distracted”, “In this period when I have to stay at home, I am worried that my college career will be permanently damaged”). The items were developed specifically for this study based on pre-existing validated questionnaires in the literature that explored similar themes in other contexts.
– *Risk perception related to COVID-19*: The adjusted Italian version [24] of the Risk Perception of Infectious Disease Questionnaire [25] was used to evaluate perceived personal susceptibility (i.e., the perceived probability of getting sick with COVID-19) and perceived comparative susceptibility (the perceived probability of getting sick with COVID-19 compared with other people of the same age and gender). Participants responded to questions using a five-point Likert-type scale ranging from 0 (not at all) to 4 (extremely). The psychometric characteristics of the original version of the Risk Perception of Infectious Disease Questionnaire are good (Cronbach’s alpha = 0.79). The questionnaire has been translated and used in many international studies [24,26,27].
– *Psychological impact of the lockdown*: A validated scale first developed for high-school students [28] and then adapted for university students was used to evaluate affective experiences related to the pandemic. The participants had to answer each item (e.g., “In this period when I must stay home, I feel well physically”; “In this period when I must stay home, I am tense and I feel tight”) on a five-point Likert-type scale ranging from 1 (not at all) to 5 (most of the time). The scale measures two affective dimensions: “negative affectivity” and “positive affectivity”. The negative affectivity scale (Cronbach’s α = 0.81) assesses the presence of feelings such as sadness, loneliness, anxiety, and guilt; it consists of 9 items, and the scores range from a low of 9 to a high of 45. The positive affectivity scale (Cronbach’s α = 0.78) assesses feelings such as energy, optimism, and joy; it comprises 6 items, and the scores range between 6 and 30. Higher scores correspond to higher levels of negative or positive affectivity.

### 2.3. Statistical Analyses

Confirmatory factor analysis (CFA) was conducted to validate the construct, validity, and reliability of the scales used in this study. The comparative fit index (CFI), Tucker–Lewis index (TLI), root-mean-square error of approximation (RMSEA), and root-mean-square residual (RMSR) were used to assess the goodness of fit. Specifically, CFI and TLI values > 0.90 [29], RMSEA values < 0.05 [30], and RMSR values < 0.08 [30] were considered to be indicative of a good fit. In addition, reliability was evaluated using Cronbach’s α, and α values > 0.70 were deemed acceptable [31].

Frequencies and percentages were used for categorical variables, while the mean (M) ± standard deviation (SD) was used for continuous variables. Multivariate analyses of variance (MANOVAs) were used to assess the association between the pandemic stage and the investigated variables (one-way between groups), as well as how sex, age, and living in a “red zone” were associated with the study outcomes (three-way between groups). A preliminary assumption check was conducted to ensure normality, linearity, homogeneity, variance–covariance matrices, and multicollinearity, but no severe violations were noted. All multivariate effects were evaluated using Wilks’ lambda.

Finally, logistic regressions were performed to predict the likelihood of reporting high levels of negative and positive affectivity during the two waves of the pandemic, and to evaluate any differences in risk and protective factors over a pandemic year. Specifically, we considered as a dependent variable the dichotomized version of the “positive affectivity” and “negative affectivity” scores (divided into high and low, assuming the median as the cutoff), and as independent variables the main sociodemographic variables and the dimensions of perception of health risks.

A statistical significance level < 0.05 was assumed at each study stage. Statistical analyses were performed using the Statistical Package for the Social Sciences (SPSS) version 25.0 (IBM Corporation, Armonk, NY).

## 3. Results

### 3.1. Psychometric Characteristics of the Scales

According to the CFA results, the goodness of fit was satisfactory for all scales. Regarding the model for the scale of “negative affectivity”, the chi-squared statistic was not statistically significant (χ^2^(9) = 16.5; *p* = 0.057), and the other values were indicative of a good fit of the model (RMSEA = 0.035; SRMR = 0.023; CFI = 0.986; TLI = 0.976). Similar results were obtained for the model of the “positive affectivity” scores (χ^2^(9) = 29.6; *p* < 0.001; RMSEA = 0.059; SRMR = 0.026; CFI = 0.981; TLI = 0.968).

### 3.2. Sociodemographic Characteristics of the Sample

The study population comprised 1230 Italian university students; 654 answered the questionnaire during stage 1 of the study; of these, 576 participated in stage 2. In both phases of the study, females were overrepresented. In the first stage, of the 654 participants, 72.8% were female and 27.2% were male; similarly, in the second stage, of the 576 participants, 78% were female and 22% were male.

The majority of the sample were undergraduate students (434 (66.3%) and 389 (67.6%)). In addition, the vast majority of the sample did not live in cities or towns declared to be “red zones” (513 (78.4%) and 531 (92.2%), *p* < 0.001). A detailed description of the study group is presented in Table 1.

### 3.3. Experiences and Opinions of Distance Learning

Regarding the opinions of the university students in our sample on the resumption of academic activities, significant differences were found between the first and second phases of the pandemic. Specifically, university students tended to be more supportive of resuming in-person educational activities during the second phase (χ^2^(1) = 12.38, *p* < 0.001). Furthermore, compared to the first phase of the pandemic, students were more in disagreement with continuing distance academic activities (except for exams) in the second phase (χ^2^(1) = 41.60, *p* < 0.001). Instead, they were more in favor of continuing all online educational activities, except for graduation ceremonies (χ^2^(1) = 16.13, *p* < 0.001). Regarding the influence of sociodemographic variables on these opinions, students in the 18–20 age group disagreed more with the possibility of continuing with all academic activities in the online mode (χ^2^(4) = 16.94, *p* = 0.002). Furthermore, off-campus students were more disagreeable with continuing online activities, except for profit exams (χ^2^(1) = 4.18, *p* = 0.04). Finally, undergraduate students were more in favor of continuing with online activities, except for profit exams (χ^2^(3) = 13.73, *p* = 0.003).

**Figure 1:**
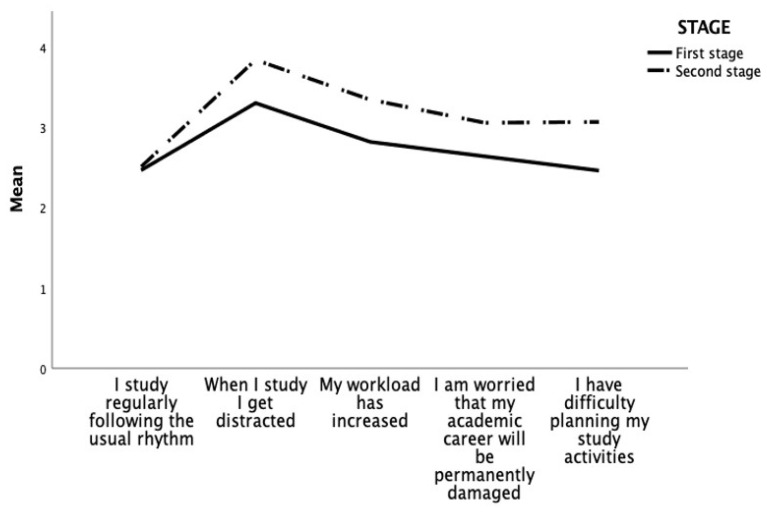
Differences in the impact of distance learning in the two waves of the pandemic.

Regarding the impact of university closures and the transition to distance learning on the wellbeing of university students, the one-way between-groups MANOVA showed a significant main effect of the pandemic stage (F_4, 1225_ = 41.10, *p* < 0.001, η^2^ = 0.12). Specifically, students reported being more distracted (*p* < 0.001), having a heavier workload (*p* < 0.001), being more concerned that their academic career could be irreparably damaged (*p* < 0.001), and having greater difficulty planning study activities (*p* < 0.001) during the second phase of the pandemic than the first (Figure 1).

Analyzing the impact of sociodemographic variables, the two-way between-groups MANOVA showed that the multivariate test of sex and age on university experience during the pandemic was statistically significant. Specifically, there was a significant main effect of age (F_16, 3718_ = 3.04, *p* < 0.001, η^2^ = 0.01), but not of sex (F_4, 1217_ = 1.57, *p* = 0.18, η^2^ = 0.005). The multivariate interaction of sex by age was significant, but the effect size was small (F_16, 3718_ = 1.95, *p* = 0.01, η^2^ = 0.006).

Considering the results separately, a statistically significant main effect of age was found on distraction during study and preoccupation with the university career. Students aged 18–20 and 21–23 reported being more distracted while studying and worried more that their university career might be harmed than older students (*p* < 0.001). The interactions of sex by age on distraction during study and preoccupation with the university career were statistically significant. Younger female students were more distracted during study activities (*p* = 0.006) and were more concerned that their academic careers could be compromised due to the pandemic (*p* < 0.001).

### 3.4. Perception of Health Risks and Fear of COVID-19

**Figure 2:**
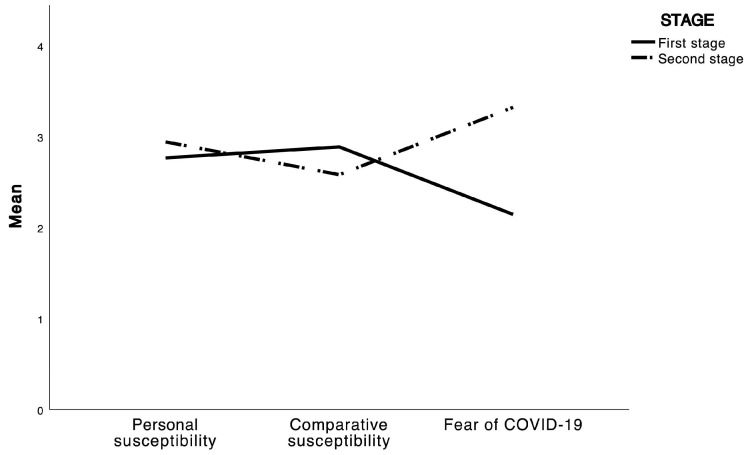
Differences in perceived health risks and fear of COVID-19 in the two pandemic waves.

The one-way between-groups MANOVA showed a significant main effect of the pandemic stage on health risk perception and fear of COVID-19 (F_3, 1174_ = 171.39, *p* < 0.001, η^2^ = 0.30). Specifically, during the second stage of the pandemic, university students in our sample reported a higher personal susceptibility and anxiety for COVID-19 and a lower comparative susceptibility (Figure 2).

In addition, a three-way between-groups MANOVA to investigate the three-way interaction of sex, age, and living in a “red zone” on health risk perceptions and fear of COVID-19 was conducted. There were main effects of age (F_12, 3058_ = 2.51, *p* = 0.003, η^2^ = 0.009) and sex (F_3, 1156_ = 3.18, *p* =0.02, η^2^ = 0.008), but not of living in a “red zone” (F_3, 1156_ = 0.40, *p* = 0.75, η^2^ = 0.001). The interaction effects were not statistically significant. Specifically, female students reported a higher perceived risk and fear of COVID-19 than male students. Furthermore, during the first wave of the pandemic, students in the 21–23 age group reported higher personal susceptibility scores than students in the other age groups; similarly, during the second phase, they reported higher personal and comparative susceptibility scores (Table 2).

### 3.5. Psychological Wellbeing of University Students during the Pandemic

**Figure 3:**
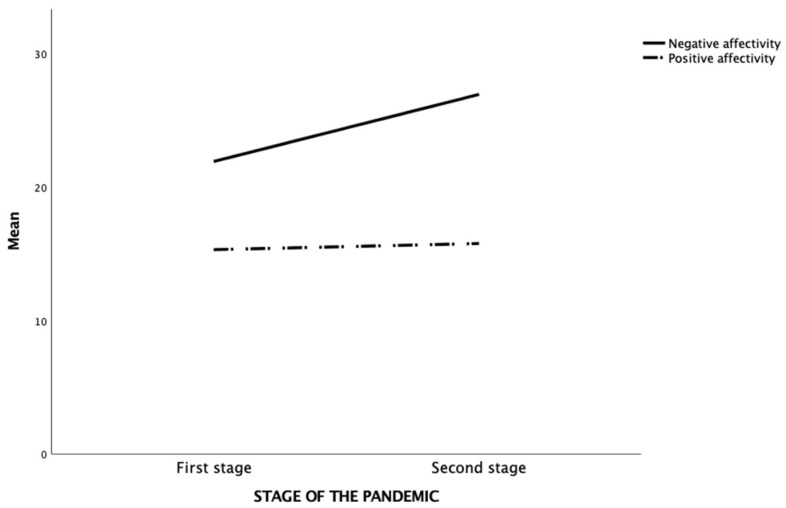
Differences in positive and negative affectivity scores in the two pandemic waves.

The one-way between-groups MANOVA showed a significant main effect of the pandemic stage on the positive and negative affectivity of university students (F_2, 1227_ = 132.33, *p* < 0.001, η^2^ = .18). In particular, a significant increase in negative affectivity scores (*p* < 0.001) and a slight increase in positive affectivity scores (*p* = 0.03) was observed in the second wave of the pandemic compared to the first (Figure 3).

A three-way between-groups MANOVA to investigate the three-way interaction of sex, age, and living in a “red zone” on positive and negative affectivity was conducted. There were main effects of sex (F_2, 1209_ = 7.31, *p* = 0.001, η^2^ = 0.01) and age (F_8, 2418_ = 2.04, *p* = 0.03, η^2^ = 0.007), but not of living in a “red zone” (F_2, 1209_ = 0.94, *p* = 0.39, η^2^ = 0.002). The interaction effects were not statistically significant.

As shown in Table 3, female students reported higher negative affectivity scores (*p* < 0.001) and lower positive affectivity scores (*p* = 0.01) than males. Furthermore, students aged 18–20 and 21–23 reported higher negative affectivity than older students (*p* < 0.001).

As a final step in our analysis, we performed a logistic regression model to identify the main predictors of positive and negative affectivity after one year of the pandemic. Sociodemographic variables, perceived health risks, and adherence to restrictive government measures were used as independent variables, while positive and negative affectivity scores were used as dependent variables.

Regarding negative affectivity, the full model containing all predictors was statistically significant (χ^2^ (15) = 198.09, *p* < 0.001). Furthermore, the model explained between 31.5% (Cox–Snell R-squared) and 42.6% (Nagelkerke R-squared) of the variance in negative affectivity and correctly classified 77.5% of cases. According to the results, the female gender increased the odds of experiencing high levels of negative affectivity after one year of the pandemic by 3.48. The concern about the university career was another significant risk factor for high levels of negative affectivity after one year of the pandemic (OR = 2.27; 95% CI = 1.90, 2.70; *p* < 0.001). Compliance with the pandemic restrictions (OR = 0.72; 95% CI = 0.57, 0.90; *p* = 0.005), family support (OR = 0.69; 95% CI = 0.57, 0.85; *p* < 0.001), and good comparative susceptibility (OR = 0.66; 95% CI = 0.46, 0.92; *p* = 0.01) were significant protective factors.

Regarding positive affectivity, the full model containing all predictors was statistically significant (χ^2^ (15) = 110.26, *p* < 0.001). Furthermore, the model explained between 19% (Cox–Snell R-squared) and 25.3% (Nagelkerke R-squared) of the variance in positive affectivity and correctly classified 68.3% of cases. Specifically, the main predictors of low levels of positive affectivity after one year of the pandemic were concern about university career (OR = 0.61; 95% CI = 0.53, 0.70; *p* < 0.001) and female gender (OR = 0.50; 95% CI = 0.30, 0.81; *p* = 0.005). Comparatively, the main predictors of high levels of positive affectivity were compliance with the restrictions against the pandemic (OR = 1.39; 95% CI = 1.14, 1.70; *p* = 0.001) and family support (OR = 1.22; 95% CI = 1.03, 1.45; *p* = 0.02).

## 4. Discussion

This study aimed to compare the experiences of a large sample of Italian university students during the first year of the COVID-19 pandemic. Specifically, we evaluated differences in students’ opinions on distance education and its impact on their wellbeing and academic experience, as well as the psychological effects of the pandemic during the first two waves of the pandemic. Additionally, the study investigated the changes in health risk perceptions related to COVID-19 in the second wave compared to the first.

Our results underline the significant impact of the pandemic and the transition to distance learning on the wellbeing of university students. Compared to the initial period of the COVID-19 lockdown, the students complained of a more significant workload, greater difficulties studying, and more concerns for their university careers and future during the second wave of the pandemic. As expected, female and younger students reported the most significant challenges, as these were the categories most affected by the impact of the COVID-19 pandemic from a psychological and emotional point of view [15,16].

Furthermore, our results confirm the significant increase in academic workload during the second phase of the pandemic, as highlighted in other studies [15]. These findings require particular attention, highlighting the need to reorganize post-pandemic university activities properly. In particular, it is essential to plan strategies for organizing university teaching that enables students not to be overloaded and to study without high stress.

University students in our sample were very concerned about their careers after one year of the COVID-19 pandemic, confirming the highly traumatic impact of the pandemic in a group like that of university students, who are already confronted with feelings of uncertainty about studies, job security, and financial stability [32]. Specifically, our results showed that uncertainty and worry about the university career and how it might be affected in the future by the pandemic was a significant risk factor for negative psychological experiences. This uncertainty, related explicitly to COVID-19, adds to the uncertainty that typically characterizes the transition from adolescence to adulthood, and is likely to exacerbate it with further negative psychological consequences, as already pointed out in several studies on the topic [14,33,34].

Despite the negative impact of the new university teaching format, most of the sample still considered it appropriate to continue online education in the subsequent phases of the pandemic to avoid the resurgence of infections. However, the percentage of students who would like to resume regular in-person activities had increased significantly after one year. In particular, students aged 18–20 disagreed more with continuing all online activities, probably due to their greater need for sociality and the more significant difficulties in organizing their study with distance learning.

Our results indicated significant psychological effects of the pandemic period on university students. A considerable increase in scores related to negative affectivity was observed after one year of the pandemic. As reported by similar studies, these data contrast with data concerning the general population, in which psychological distress decreased slightly in the second wave of the pandemic [32]. This discrepancy can be explained by the fact that, compared to the general population, university students experienced a particular and challenging stage of development, so they suffered more from the effects of the COVID-19 pandemic, as it affected the different areas of their daily lives much more markedly [16,17,32].

These results are consistent with other studies examining university students’ mental health during the pandemic. For example, a recent study at the University of Valladolid (Spain) showed higher anxiety, stress, and depression among students and administrative staff [35]. Similarly, another recent study in Greece reported the detrimental psychological effects of quarantine on university students’ mental health [36]. In addition, a cross-sectional web-based survey conducted on 476 university students living in Bangladesh during the COVID-19 pandemic confirmed that university students experienced increased depression and anxiety.

However, despite the significant increase in negative affectivity scores in the second phase of the pandemic, there was also a slight increase in positive affectivity scores. This result confirms that positive and negative affectivity are two independent dimensions that do not necessarily vary similarly [37], and this highlights that university students can rely on protective factors that maintain a good level of positive affectivity. Among these, family support was associated with better levels of positive affectivity, as has already been widely noted in the literature [38,39].

The university students in this study showed a moderate perception of the risk of contracting COVID-19. However, a significant increase in personal susceptibility and a decrease in comparative susceptibility could be observed after one year. Fear and concern about the risk of contracting the disease were also significantly higher in the second phase of the pandemic. These data are particularly interesting because they show increased awareness of the threat posed by COVID-19 among university students, resulting in increased concern and perception of personal risk related to the disease. However, the decrease in perceived risk relative to others after one year is another finding to consider. This discrepancy has already been widely confirmed in the literature on health risk perceptions, showing that an individual’s risk perceptions about the same disease can be simultaneously pessimistic and optimistic, depending on whether it is intended in absolute or comparative terms [40,41]. According to the literature, the implications may be different. In fact, according to some studies, excessive optimism may lead to lower motivation to adopt preventive behaviors [42,43]. On the contrary, other studies say that unrealistic optimism may be associated with positive health outcomes [40,44]. In the case of our research, university students tended to be more pessimistic about their personal risk but more optimistic about the risk perceived in comparative terms than people of the same age and sex. However, this discrepancy does not appear to have affected the adherence to restriction measures to contain infections, which did not change significantly one year later.

According to our findings, female students expressed a higher perceived susceptibility and tended to be more concerned about the disease than males. These results are also consistent with data from the literature on university students’ perceptions of risk during pandemics [45,46]. In this regard, a study by Akan et al. [47] on university students’ knowledge and attitudes toward the influenza A/H1N1 pandemic demonstrated that 40.5% of the participants perceived their risk as “moderate” and that the risk perception of men was significantly lower than that of women, as reported in this study.

Another interesting finding is that students in the age group 21–23 years reported higher personal susceptibility scores than students in the other age groups; similarly, during the second phase, they reported higher personal and comparative susceptibility scores. Contrary to the literature showing that health risk perception increases with age [48,49], younger students in our sample had higher risk perception than older students. A possible interpretation is that the younger population, in general, appears to have been more negatively affected by the COVID-19 pandemic [50,51], and that university students in the initial stages of their careers have more doubts and uncertainties about their future [52,53,54], which were dramatically amplified by such an unexpected and unpredictable event as the pandemic [16,17]. Consequently, this more significant impact may be associated with a higher risk perception than that of older students.

Finally, we evaluated the main predictors of positive and negative affectivity one year after the pandemic’s outbreak, drawing the identity of the university students most at risk of developing psychological distress due to the COVID-19 pandemic. The main risk factors for high negative and low positive affectivity after one year of the pandemic were female gender and concern about university career. In contrast, compliance with the restrictions against the pandemic, family support, and good comparative susceptibility were significant protective factors.

One of the main strengths of this study is the large sample of university students enrolled over a pandemic year to provide a comprehensive overview of the impact of COVID-19 on the experience of university students in Italy. Furthermore, compared to other studies already published on the topic, we analyzed a few investigated variables that may influence the psychological experiences of university students during the pandemic, such as perception of health risk. However, this study also has limitations that should be considered. First, convenience sampling by sharing an online questionnaire did not balance the sample for sociodemographic variables, such as sex and age. Second, using an online questionnaire may have created bias, as it was not possible to ensure the participants’ accuracy in answering the questions. Finally, the cross-sectional design of this study did not allow for changes in psychological experiences within a year. For this purpose, a longitudinal design would have allowed for a better understanding of the effects of COVID-19 on university students. Still, applying this study design was difficult due to the restrictions caused by the pandemic.

## 5. Conclusions

In conclusion, this study shows that the university period is a critical phase in individual development and can be affected by stressful and unexpected events such as the COVID-19 pandemic. In fact, both the pandemic and the related restrictive measures seemed to have a strong negative impact on the wellbeing of university students in our sample during the first two waves in Italy. In particular, the results of this study confirm that, over a pandemic year, university students in our sample reported increasing levels of negative affectivity, study difficulties, and academic career concerns. However, we identified protective factors that mitigated the psychosocial effects of the two waves of the pandemic, such as support from family and friends. From this perspective, the end of the acute health emergency period cannot exempt universities from continuing to consider students’ wellbeing and meet their academic and personal needs. In this regard, the growing awareness of the importance of university students’ mental health and the significant impact that it has on academic success should encourage universities to implement or create, where they do not exist, specific services and broad-based interventions to provide adequate psychological support to the most distressed students.

## Figures and Tables

**Table 1:** Sociodemographic characteristics of the sample.

		Stage 1 (n = 654)		Stage 2 (n = 576)	
		n	%	n	%
Sex	Female	476	72.8	449	78.0
	Male	178	27.2	127	22.0
Age (years)	18–20	151	23.1	100	17.4
	21–23	328	50.2	344	59.7
	24–26	122	18.7	90	15.6
	27–29	22	3.4	21	3.6
	30+	31	4.7	21	3.6
Type of university course	Undergraduate	434	66.3	389	67.6
	Postgraduate	133	20.4	122	21.2
	Out-of-course	85	13.0	65	11.3
	PhD	2	0.3	0	0.0
Resident in the university town	No	305	46.6	299	51.9
	Yes	349	53.4	277	48.1
“Red zone”	Yes	141	21.6	45	7.8
	No	513	78.4	531	92.2
Size of the household	1	31	4.7	17	3.0
	2	72	11.0	72	12.5
	3	178	27.2	158	27.4
	4	255	39.0	230	39.9
	> 4	118	18.0	99	17.2
Personally contracted COVID-19	Yes	1	0.0	34	5.9
	No	653	86.6	493	85.6
	Uncertain	0	13.3	49	8.5

**Table 2:** Means and standard deviations of health risk perception and fear of COVID-19 scores in both stages of the study.

		Personal Susceptibility		Comparative Susceptibility		Fear of COVID-19	
		Stage 1 (n = 654)	Stage 2 (n = 576)	Stage 1 (n = 654)	Stage 2 (n = 576)	Stage 1 (n = 654)	Stage 2 (n = 576)
Sex	Male	2.62 ± 0.78	2.76 ± 0.85	2.86 ± 0.59	2.39 ± 0.85	2.02 ± 0.84	2.89 ± 1.23
	Female	2.82 ± 0.79	2.99 ± 0.70	2.89 ± 0.54	2.63 ± 0.68	2.19 ± 0.86	3.43 ± 1.24
Age	18–20	2.62 ± 0.84	2.85 ± 0.63	2.87 ± 0.59	2.40 ± 0.70	2.13 ± 0.89	3.30 ± 1.33
	21–23	2.82 ± 0.77	3.02 ± 0.74	2.91 ± 0.54	2.65 ± 0.70	2.11 ± 0.82	3.35 ± 1.24
	24–26	2.74 ± 0.84	2.85 ± 0.80	2.82 ± 0.51	2.54 ± 0.78	2.20 ± 0.89	3.20 ± 1.30
	27–29	2.73 ± 0.70	2.47 ± 0.96	2.82 ± 0.39	2.30 ± 0.80	2.32 ± 0.99	3.38 ± 1.35
	>30	2.63 ± 0.70	2.86 ± 0.65	2.60 ± 0.73	2.46 ± 0.76	2.16 ± 0.89	3.19 ± 1.03
“Red zone”	Yes	2.74 ± 0.76	2.93 ± 0.76	2.87 ± 0.56	2.58 ± 0.73	2.16 ± 0.87	3.11 ± 1.45
	No	2.84 ± 0.89	3.05 ± 0.60	2.94 ± 0.55	2.60 ± 0.73	2.07 ± 0.83	3.33 ± 1.24

**Table 3:** Means and standard deviations of positive and negative affectivity scores in both stages of the study.

		Positive Affectivity		Negative Affectivity	
		Stage 1 (n = 654)	Stage 2 (n = 576)	Stage 1 (n = 654)	Stage 2 (n = 654)
Sex	Male	15.79 ± 3.52	17.25 ± 4.28	19.69 ± 6.07	22.18 ± 8.04
	Female	15.10 ± 3.74	15.33 ± 3.72	22.73 ± 6.91	28.28 ± 7.67
Age	18–20	15.21 ± 3.59	15.78 ± 4.25	22.47 ± 6.64	27.65 ± 7.31
	21–23	14.94 ± 3.84	15.58 ± 3.79	22.26 ± 6.73	27.20 ± 8.22
	24–26	15.81 ± 3.49	16.02 ± 4.01	21.37 ± 7.09	26.16 ± 8.20
	27–29	16.31 ± 2.80	15.14 ± 4.17	19.95 ± 6.22	28.14 ± 8.30
	>30	16.58 ± 3.55	18.00 ± 3.59	18.87 ± 7.28	21.38 ± 8.93
“Red zone”	Yes	14.56 ± 3.94	15.02 ± 3.87	23.40 ± 7.50	29.62 ± 8.45
	No	15.49 ± 3.60	15.81 ± 3.93	21.49 ± 6.58	26.71 ± 8.09

## Data Availability

The data presented in this study are available upon request from the corresponding author. The data are not publicly available due to departmental policy.
